# Education

**Published:** 1862-05

**Authors:** 


**EDUCATION** is literally "a leading from;" it may be from ignorance to knowledge; from vice to virtue. This is education in a good sense. But we may be educated, "led from" virtue to vice; from truth to error; so that in its largest acceptation, education is the being "led from" an old state or habit of thought, feeling, and action, into some other state of thought, feeling, and action. But we may educate the body as well as the mind and heart; we may educate it from a sickly, diseased condition into robust health and manly vigor; we may also bring it from these down to rottenness and imbecility. A true and comprehensive education, in reference especially to our children, is that which leads out the bodily powers to greater strength; the mental to greater activities; the moral to greater purity, beneficence, and nobility—all these should be done at the same time, although in different degrees; all should be attended to during the period of youth. The moral teachings should commence with the earliest infancy; the physical, as soon as there is bodily locomotion; the mental, meaning thereby the literary, not earlier than the completion of the sixth year, not even to the extent of learning the alphabet, or repeating by "rote;" mere mechanical memorizing. This brain education is specially advised in reference only to children whose situation in life allows them to study until they are twenty-one. The children of the poor—those who must go to work and earn something—can with safety begin at the age of three or four years, for three reasons;

First. They are out in the open air nearly all the time during daylight.

Second. Their food is plain and not over-abundant.

Third. The early necessity that they should do something for a living does not allow time for special brain disturbance; and any slight tendencies in that direction would be counteracted and repaired by the constant muscular activities necessary to their condition. But those children who will have nothing to do but "get their education," up to the day of entering their twenty-first year, ought to do nothing for the first third of that period, but to eat and sleep and play out of doors from morning till night all the year round, except when rain, sleet, or snow are falling. It is the exercise daily, "regardless of the weather," which works so many, almost miracles, in the renovation of human health.

The vanity of parents is fed by the "smartness" of their children; but early ripe, early ruined, may be said of all precocities. If not actually ruined, there is almost in all cases a sudden "giving out" of the mental powers, and the prodigy of yesterday is the mediocre of to-day, and the *non compos mentis* of to-morrow.

